# Francisella tularensis as the cause of protracted fever

**DOI:** 10.1186/s12879-020-05051-1

**Published:** 2020-05-07

**Authors:** Lukas Antonitsch, Gerhard Weidinger, Gerold Stanek, Mateusz Markowicz

**Affiliations:** 1 Innere Medizin, Gastroenterologie und Hepatologie, Landesklinikum Wiener Neustadt, Landeskliniken Holding, Corvinusring 3-5, 2700 Wiener Neustadt, Austria; 2 Institute for Hygiene and Applied Immunology, Center for Pathophysiology, Infectiology and Immunology, Medical University of Vienna, Kinderspitalgasse 15, 1090 Vienna, Austria

**Keywords:** Tularemia, Francisella tularensis, Typhoid tularaemia, Pulmonary tularaemia, Fever of unknown origin,re-emerging disease, Austria

## Abstract

**Background:**

Tularemia, a re-emerging, potential life threatening infectious disease, can present itself with nonspecific clinical symptoms including fever, chills and malaise. Taking a detailed history of exposure and a highly raised index of clinical suspicion are necessary to take the appropriate diagnostic and therapeutic steps in this setting. Here, a case report of typhoid tularaemia is presented.

**Case presentation:**

A 53-year old male forester and farmer with protracted fever, abdominal pain, diarrhoea and loss of weight, who experienced productive cough and a pulmonary infiltrate later in the course of disease, was admitted for further investigation. Tularaemia was suspected only owing to history and confirmed by serologic testing more than three weeks after the beginning of the symptoms. The initial antibiotic therapy with ceftriaxone/doxycycline was switched to ciprofloxacin, resulting in the resolution of fever and symptoms.

**Conclusion:**

Tularaemia has to be considered as a differential diagnosis in febrile patients, even more in cases with protracted fever. Since tularaemia is expanding geographically, involving more animal hosts and causing larger outbreaks, clinicians have to be aware of this potentially fatal disease.

## Background

Tularemia is a rare but potentially life threatening zoonosis, and it is well recognized as a (re-) emerging disease in Austria [1–3], as well as in other European countries [4–7]. There are existing several clinical forms, of which most depend on the site of entrance to the human body; however several ways of transmission can result in different clinical forms [5, 8]. The major clinical forms of presentation are the ulceroglandular, oculoglandular, glandular, oropharyngeal, pneumonic and typhoid form.

Due to the fact that this rare disease can present itself with nonspecific clinical symptoms including fever, chills and malaise only (“typhoid form”), taking a detailed exposure history and having a highly raised index of clinical suspicion are necessary to take the appropriate diagnostic and therapeutic steps [5, 8]. The clinical presentation of our patient is consistent with a typhoid form of tularaemia, secondarily switching to the pulmonary form.

## Case presentation

A 53-year old male forester and farmer was admitted with protracted fever up to 39 °C, abdominal pain, diarrhoea without vomiting, productive cough and loss of weight (8 kg, see below).

The complaints had started three weeks earlier, with epigastric pain followed by fever and chills, therefore he sought medical attention early in the course of his disease in one of the two existing em departments (one of which is the department of cardiology) of our non-academic tertiary hospital in lower Austria. He was diagnosed with viral gastroenteritis and sent home with supportive measures; follow up with his general practitioner (GP) was suggested.

About two weeks later the complaints had persisted; moreover, he suffered from productive cough and mentioned loss of 8 kg of body weight. Following the advice of his GP, a chest x-ray has been carried out, which was unremarkable. The patient presented again at our hospital, this time at the emergency department of our gastroenterology unit (there is no general emergency department at our hospital at the moment). A detailed history was taken and he was admitted for further investigation and treatment two days later.

The patient had an unremarkable medical history apart from a mild Helicobacter-associated gastritis which had been treated a few months before and some previous respiratory infections. He was a non-smoker, with low to moderate alcohol consumption. He denied journeys to foreign countries in the recent past; he had never left Europe but had visited Greece and Croatia, in 2017 and 2016, respectively. When he was asked about his profession, a detailed history of exposure to zoonotic pathogens was carried out. He denied contact with wild animals or slaughter in the last months but reported woodwork in the forest for the last 6 weeks. He was not aware of any tick bites or injuries.

At admission, he appeared to be neither septic nor jaundiced; there was no lymphadenopathy or rash. Heart and lungs were clear to auscultation; abdominal examination showed no abnormality apart from hyperactive bowel sounds. Heart rate and blood pressure were within normal limits.

Laboratory results showed elevated leucocyte count (13/nL), platelet count (560/nL) and C-reactive protein (12 mg/dL). Other remarkable results were as follows: gamma-glutamyl transferase (gamma GT) 108 U/L, bilirubin 1.5 mg/dL, the remaining liver function tests and kidney function tests were within normal limits, as was the procalcitonin. A summary of laboratory test results and cutoff values is presented in the table (see Table 1). A chest x-ray showed a discrete pulmonary infiltrate at the right upper lobe, but no hilar enlargement.

**Table 1:** Results and cut off values of laboratory examination

	Cutoff values	21.04.2019	24.04.2019	26.04.2019	29.04.2019	19.06.2019	17.05.2019	01.07.2019
Hemoglobin (g/dL)	13.5–17.2	14.9	14.2	13.3	13.7	15.4		15.8
Wbc (G/L)	3.90–10.20	13.86	11.74	9.96	8.95	7.42		7.74
CRP (mg/dL)	0–0.5	12.0	15.0	9.8	2.8	0.2		0.3
Creatinine (mg/dL)	0.7–1.2	1.3	1.2	1.1	–	0.9		0.9
ASAT/GOT (U/L)	5–50	21	21	13	–	27		22
Gamma –GT (U/L)	10–60	108	101	86	–	36		43
Bilirubin (mg/dL)	0–1.2	1.5	1.6	0.9	–	2.0		1.3
*F. tuIarensis* IgM ELISA (U/mL)	10–15		145,3				322	
*F. tuIarensis* IgG ELISA (U/mL)	10–15		52,6				91	
*F. tularensis* agglutination titre	< 1:80		1:80				1:640	

Blood cultures were drawn and serum was sent to a reference laboratory for antibody testing for anaplasmosis, rickettsiosis, babesiosis, Q fever, brucellosis, and tularaemia. Moreover, infection with *Rickettsia* spp. and Coxiella burnetti was excluded by PCR. Tuberculosis was ruled out by a negative interferon gamma release assay (Quantiferon – QFT PLUS, Qiagen GmbH, Germany). Empiric antibiotic therapy was initiated with intravenous ceftriaxone 2 g bd and oral doxycycline 200 mg qd. The patient recovered quickly, fever, cough and abdominal complaints resolved. During the course, he recognized mild pharyngitis. A control chest x-ray didn’t show any infiltrates.

Serologic tests for tularemia were performed with an agglutination assay (CCPro, Oberdorla, Germany) and ELISA (Virion/Serion, Würzburg, Germany). The antibody titre was 1:80 in the agglutination assay which was classified as negative and the ELISA was positive for IgG (52,6 U/mL, positive cutoff > 15 U/mL) and IgM (145,3 U/mL, positive cutoff > 15 U/mL). The other serologic tests and blood cultures (after 7 days of cultivation, however) were negative, therefore an infection with *Francisella tularensis* was suspected. The therapy was switched to oral ciprofloxacin 500 mg bd to complete a 3-week course and the patient was discharged. In order to confirm the infection, serum was sent to the same laboratory 4 weeks later by his GP for a repeated serologic testing. The agglutination assay was positive at the titre of 1:640. The ELISA results changed to 91 U/mL for IgG and 322 U/mL for IgM.

## Discussion and conclusions

There are six major clinical manifestations of tularaemia, some of which are more easily to recognize (ulceroglandular, glandular, oculoglandular), whereas others resemble other, more common diseases (pharyngeal, pulmonary) or present with nonspecific symptoms (typhoid) [5, 8]. Tularaemia is an important cause of fever of unknown origin [8].

Because the initial symptoms were gastrointestinal, apart from malaise and fever, and the first chest x-ray (carried out on an outpatient basis) was unremarkable, this case represents the typhoid form with secondary pulmonary tularaemia, rather than primary pulmonary disease, even though the most likely way of transmission was by inhalation of dust during the woodwork in the forest.

In several European countries (re-)emergence of tularaemia has been recognized [1–7]. In addition, expansion of vectors and the bacteria, across Europe is expected and the involvement of a wider range of wildlife than previously thought has been reported [9].

In Austria, from 2009 to 2018, 42 cases of human tularaemia have been reported, none of which were fatal. From 2014 to 2018, 40 lagomorphs have been tested for tularaemia by AGES (Austrian Agency for Health and Food Safety GmbH), 7 of which were positive, mostly from 2016 to 2018 [10]. Rural areas of Styria, lower Austria and Burgenland were recognized as hot spots when *Vulpes vulpes* was used as bio indicator, via mandibular lymph node biopsy, for tularaemia from 2007 to 2008 [11].

The diagnosis of the infection can by performed by direct identification of the pathogen. However, cultivation requires a high level security laboratory therefore serologic testing seems a more appropriate and less demanding diagnostic procedure. Moreover, prolonged cultivation can be necessary to yield growth – in our case, blood cultures were cultivated for 7 days only, which is a standard procedure in our hospital.

In conclusion, Tularaemia has to be considered as a differential diagnosis in febrile patients, even more in cases with protracted fever. Since tularaemia is expanding geographically, involving more animal hosts and causing larger outbreaks, clinicians have to be aware of this potentially fatal disease.

## Data Availability

Not applicable - Records of laboratory values as given in the text or table are saved on our hospital server. If strongly needed, they could be provided in case of publication, since this is a case report we don’t consider this as necessary.

## Abbreviations

GP: General practitioner
em department: Emergency medicine departement
